# An evolutionarily conserved SSNA1/DIP13 homologue is a component of both basal and apical complexes of *Toxoplasma gondii*

**DOI:** 10.1038/srep27809

**Published:** 2016-06-21

**Authors:** Maude F. Lévêque, Laurence Berry, Sébastien Besteiro

**Affiliations:** 1 DIMNP, UMR5235, CNRS, University of Montpellier, Montpellier, France

## Abstract

Microtubule-based cytoskeletal structures have fundamental roles in several essential eukaryotic processes, including transport of intracellular constituents as well as ciliary and flagellar mobility. Temporal and spatial organisation of microtubules is determined by microtubule organising centers and a number of appendages and accessory proteins. Members of the SSNA1/DIP13 family are coiled coil proteins that are known to localise to microtubular structures like centrosomes and flagella, but are otherwise poorly characterised. We have identified a homologue of SSNA1/DIP13 in the parasitic protist *Toxoplasma gondii* and found it localises to parasite-specific cytoskeletal structures: the conoid in the apical complex of mature and dividing cells, and the basal complex in elongating daughter cells during cell division. This protein is dispensable for parasite growth *in vitro*. However, quite remarkably, this coiled coil protein is able to self-associate into higher order structures both *in vitro* and *in vivo*, and its overexpression is impairing parasite division.

The cytoskeleton is necessary for maintaining the cell’s shape, allowing cell motility and for intracellular trafficking. Microtubules are one of the three main types of filaments constituting the cell’s cytoskeleton (together with actin and intermediate filaments). They are involved in a number of important cellular processes, including the transport of organelles and proteins, the establishment of cell polarity, ciliary and flagellar mobility, and the cell cycle-dependent formation of the mitotic spindle. These dynamic and polarised processes are depending on a structure promoting the formation and ordered arrangement of microtubules, called the microtubule organising centre (MTOC)^1^.

*Toxoplasma gondii* is a parasitic protist belonging to the phylum Apicomplexa. This group of ancient eukaryotes comprises many pathogens responsible for important diseases in both animals and humans. *T. gondii* is one of the most widespread zoonotic parasites, as it infects up to a third of the world’s population. It is the causative agent of toxoplasmosis, a disease affecting primarily immunocompromised individuals and developing fetuses^2^. The initial spread of infection is caused by a quickly multiplying form called tachyzoite. Tachyzoites are highly polarised cells that will invade a host cell and subsequently replicate intracellularly by a process termed endodyogeny, in which two daughter parasites assemble within a mother parasite^3^,^4^. This complex coordinated process depends on the function of two distinct microtubule populations: spindle microtubules and subpellicular microtubules^5^. Endodyogeny involves a closed mitosis during which spindle microtubules remain intranuclear to coordinate chromosome segregation, while juxtanuclear centrioles are found at the spindle poles and serve as the nuclear MTOC. Tachyzoites also contain another, rather unusual, MTOC localised at their very apical end and called the apical polar ring (APR)^6^. This structure is part of the so-called apical complex, one of the defining features of apicomplexan parasites, that contains a set of peculiar cytoskeletal elements^7^,^8^. The apical complex is organised around the conoid, an intriguing highly dynamic organelle, which is supposed to play a role in parasite motility and host cell invasion^8^,^9^. The conoid is a truncated cone made of about 14 curved tubulin fibers arranged into a spiral like a compressed spring^10^. Inside the conoid is a pair of short microtubules whose function is unknown, although they have been suspected to be involved in the transport of invasion-related secretory vesicles. Several ring-like structures are closely associated with the conoid: two preconoidal rings (PCR), at its distal tip, and the APR, from which 22 subpellicular microtubules radiate to give the parasite its crescent shape. These microtubules extend towards the parasite posterior end, in close association with the inner membrane complex (IMC), a peripheral membrane system composed of flattened alveolar sacs that underlie the plasma membrane^3^,^4^. The conoid is a mobile structure: during intracellular development, it is usually recessed inside the APR at the tip of the parasite, but during invasion it is extruded from the APR in a calcium-dependent fashion^11^, to form an extended point of contact with the host cell. At the posterior end of the IMC lies the basal complex, another cytoskeleton-associated compartment, constructed early during daughter cell budding for which it could play a key role, but whose precise function in mature parasites is currently unknown^8^,^12^,^13^,^14^.

Sjögren’s Syndrome nuclear autoantigen 1 (SSNA1, also known as NA14^15^) is the founding member of a new family of proteins. Parallel studies on human SSNA1 and its homologue in phylogenetically-distant green algae *Chlamydomonas reinhardtii* (‘deflagellation inducible protein of 13 kDa’, DIP13), reported as a common feature that this protein associates with microtubular structures (basal bodies, centrosome, and flagella), and is potentially important during cell division^16^. A more recent study on protist *Trypanosoma brucei* also characterised a SSNA1/DIP13 homologue that was found to co-localise with acetylated tubulin^17^. SSNA1 has been initially characterised as one of the main targets of autoantibodies in the context of Sjögren’s syndrome^18^, a relatively frequent human autoimmune disorder (about 0.5% prevalence in the population), in which the immune system primarily attacks the glands that produce tears and saliva, impairing their ability to secrete these fluids^19^. Large scale studies have suggested a potential association of SSNA1 with the centrosome^20^ and cilia components^21^ in vertebrates, but its precise cellular function and its exact implication in the establishment of the disease are currently unknown. Thus, although data suggested SSNA1/DIP13 and related proteins could have a cytoskeleton-related function that is conserved in phylogenetically-distant eukaryotes, this protein family remains largely enigmatic with respect to its structure and function.

In the present study, we describe the characterisation of the *T. gondii* SSNA1/DIP13 homologue. We show this protein has biochemical properties which are similar to those of its other eukaryotic counterparts, but it also has a peculiar localisation at the apical complex, and at the basal complex in developing daughter cells, that suggests it has evolved specialised functions in these parasites.

## Results

### Identification of a *T. gondii* SSNA1/DIP13 homologue

**Figure 1:**
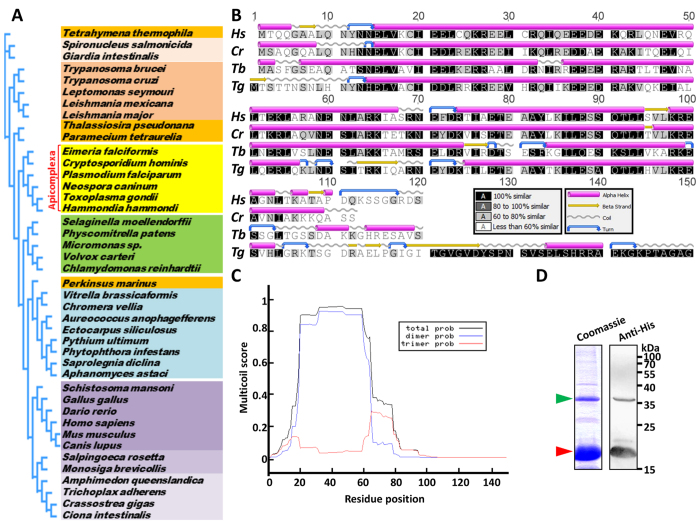
TgDIP13 is a conserved coiled coil protein. (**A**) Phylogenetic analysis of representative members of the SSNA1/DIP13 family. Apicomplexan parasites are annotated. (**B**) Alignment of *Toxoplasma gondii* DIP13 amino acids sequence (*Tg*) with previously identified eukaryotic homologues: *Homo sapiens* SSNA1/NA14 (*Hs*), green algae *Chlamydomonas reinhardtii* DIP13 (*Cr*) and parasitic protist *Trypanosoma brucei* DIP13 (*Tb*). Secondary structure predictions show most of these proteins are in the form of an α-helix. (**C**) Prediction of oligomerisation states of coiled-coil domain of TgDIP13 by the Multicoil program (http://groups.csail.mit.edu/cb/multicoil/cgi-bin/multicoil.cgi). The score for potential coiled-coils with a dimeric probability is indicated by the blue line and a trimeric probability is indicated by the red line. The sum of these two probabilities is the total probability for forming a coiled-coil, which is indicated by the black line. (**D**) Purified His-tagged recombinant TgDIP13 is detected both as a monomer (~18 kDa, red arrowhead) and a dimer (~36 kDa, green arrowhead) by Coomassie staining and immunoblot with anti-His antibody.

Homologues of SSNA1/DIP13 have been identified previously in a number of eukaryotes, including protozoa, trematode and fish^16^. We extended the analysis of the phylogenetic distribution of these proteins and confirmed their presence in a wide array of eukaryotic species (Fig. 1A), suggesting an ancestral function for the SSNA1/DIP13 family. Interestingly, all these eukaryotes have centrioles, or produce cilia/flagella at some point during their lifecycle. Conversely, these proteins seem absent from non-ciliated species and, strikingly, also from *Drosophila melanogaster* and *Caenorhabditis elegans*, both of which have cilia but build unusual centrioles^22^.

SSNA1/DIP13 homologues have been described as coiled coil proteins^15^,^16^,^17^. The *T. gondii* SSNA1/DIP13 homologue we have identified (www.Toxodb.org entry TGME49_295450) presents a C-terminal extension, but is otherwise predicted to be essentially in the form of an α-helix (Fig. 1B). Sequence analysis confirmed that this protein, that we named TgDIP13, is a putative coiled coil protein most probably forming a dimer (Fig. 1C). We expressed a His-tagged recombinant form of TgDIP13 in *Escherichia coli*, and confirmed experimentally the ability of the purified protein to associate as a dimer (Fig. 1D).

### The TgDIP13 homologue localises to the conoid

**Figure 2:**
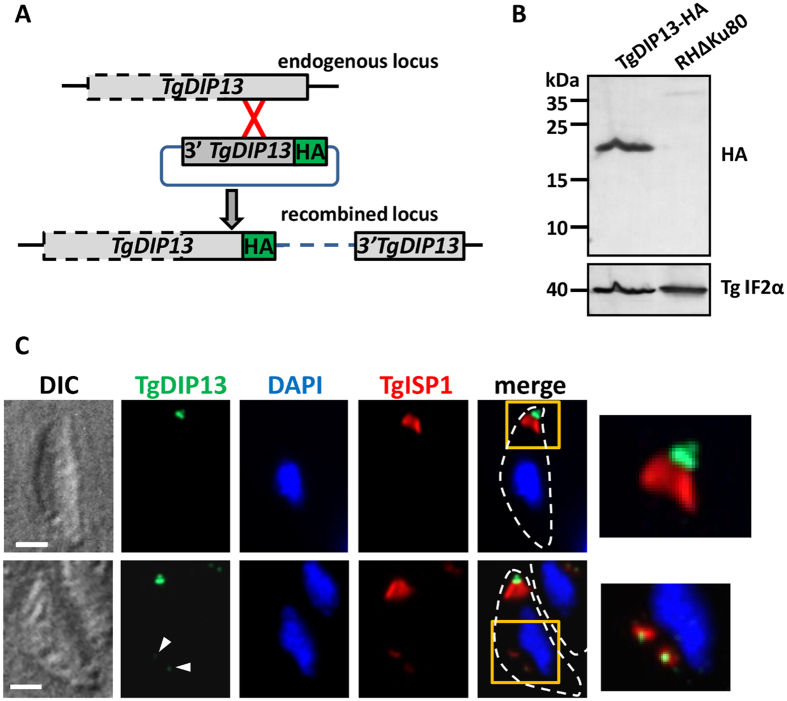
Tagging and localisation of endogenous TgDIP13. (**A**) Strategy to express an HA-tagged version of TgDIP13 from its endogenous locus. (**B**) Immunoblot analysis of the HA-tagged protein in total cell lysates from transgenic parasites after selection. *Toxoplasma* initiation factor-2 alpha (TgIF2α) was used as a loading control. (**C**) Immunofluorescence detection of HA-tagged TgDIP13 and IMC cap protein TgISP1 in intracellular parasites. Bottom series show a parasite containing nascent daughter cells. Parasite shape is outlined on the merged images. Magnifications of squared areas are presented on the right. DNA was labelled with DAPI. DIC: differential interference contrast. Scale bars represent 1 μm.

To determine the subcellular localisation of TgDIP13, its corresponding gene was modified at the endogenous locus by single homologous recombination (Fig. 2A), to express a C-terminal hemagglutinin (HA) epitope-tagged version of the protein in *Toxoplasma* tachyzoites (TgDIP13-HA). In accordance with its predicted amino acids sequence, the tagged version of TgDIP13 has an apparent molecular mass of ~18 kDa, as verified by immunoblot (Fig. 2B). Immunofluorecence assay (IFA) revealed that this protein localises to the very apical end of the parasites (Fig. 2C), essentially above the apical cap portion of the IMC labelled with ISP1^23^. This localisation was likely corresponding to the conoid. However, this apical labelling was not uniform: rather, it appeared as ring/band and a more apical dot, with dark space in between (Fig. 2C), suggesting a localisation of TgDIP13 at two distinct subcompartments of the organelle. IFA also revealed that TgDIP13 is produced early during parasite division, as it can be observed in daughter cells when their IMC has just started to emerge (Fig. 2C). Closer analysis and quantification of the TgDIP13 signals in non-dividing cells showed that, besides the conoid localisation systematically present in all parasites, the protein was also occasionally found to form an additional, but not necessarily apical, punctate structure (in about 1/3 of intracellular parasites), and, more rarely, as an intriguing fiber-like structure (in ~5% of intracellular parasites) (Figure S1).

**Figure 3:**
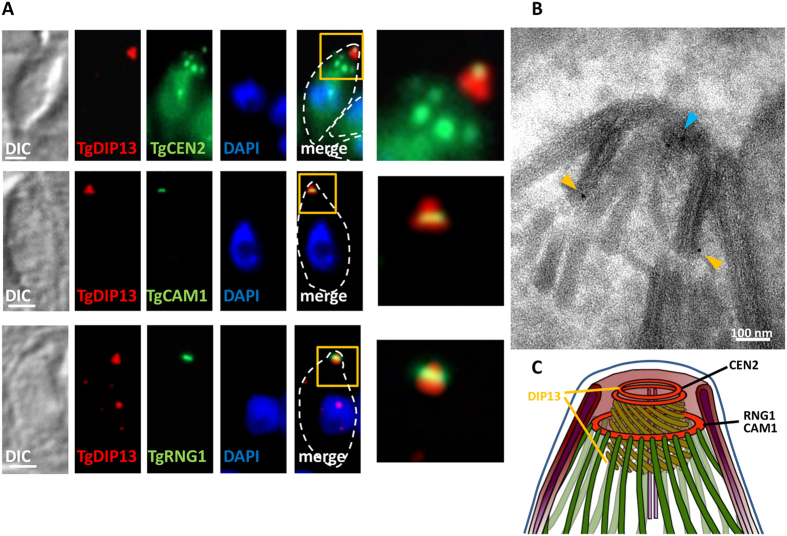
TgDIP13 localises to two distinct subregions of recessed conoids. (**A**) Co-staining between HA-tagged TgDIP13 and apical complex markers TgCEN2, TgCAM1 and TgRNG1. DNA was labelled with DAPI. DIC: differential interference contrast. Parasite shape is outlined on the merged images. Magnifications of squared areas are presented on the right. Scale bars represent 1 μm. (**B**) Immunoelectron microscopy on Triton X-100-extracted cytoskeleton shows HA-tagged TgDIP13 localises both to the very apical part of the conoid (blue arrowhead) and at its base (orange arrowheads). (**C**) Schematic representation of the localisation of TgDIP13 and other apical complex markers in a recessed conoid conformation.

The apical complex comprises not only the conoid, but also a number of closely associated structures such as the polar rings. To get more insights into the precise localisation of TgDIP13 at the apical complex, we performed IFA by co-staining with markers of these subdomains (Fig. 3A). *T. gondii* centrin 2 (TgCEN2) in addition to its centriolar localisation, is found in peripheral annuli at the apical portion of the parasite body, and at the PCR at the extreme apical end of the parasites^8^. We found that the very apical dot stained with TgDIP13 colocalised extensively with TgCEN2-labelled PCR. The larger ring-like signal labelled with TgDIP13 was found to be systematically posterior to TgCAM1 and TgRNG1, which are proteins localising near the middle part of the conoid, or at the APR, respectively^8^,^24^. To overcome the resolution limits of light microscopy and gain additional information on TgDIP13 localisation in the apical complex substructures, we also performed immuno-electron microscopy using anti-HA antibodies (Fig. 3B). This analysis confirmed TgDIP13 associates with a very apical structure above the conoid, presumably the PCR, as well as the base of the organelle (Fig. 3B,C).

The conoid is a mobile structure whose extrusion is depending on a calcium flux^11^. We used a calcium ionophore on freshly egressed extracellular parasites, before fixation and IFA of TgDIP13 together with the aforementioned apical complex markers (Figure S2A). In these conditions, TgDIP13 was found exclusively as a single apical signal co-localising with TgCEN2, and anterior to TgCAM1 and TgRNG1. This is consistent with an association of TgDIP13 with the upper part of the conoid (Figure S2B), however the fate of the basolateral signal observed in recessed conoid conditions is unclear after extrusion of the organelle.

### TgDIP13 is also present at the basal complex of elongating daughter cells during cell division

During cell division of *T. gondii* tachyzoites, the earliest events are the duplication of the centrosomes and of the Golgi apparatus^25^. The earliest nascent components of the apical complex, such as TgRNG2, are recruited soon after centrosome duplication^26^, while other proteins are recruited much later to the structure, in the nearly mature daughter cells^24^. We performed IFA on dividing parasites using centrosomal marker centrin 1, and found that TgDIP13 appears in daughter cells in close proximity to the centrosomes after they have duplicated (Figure S3).

**Figure 4:**
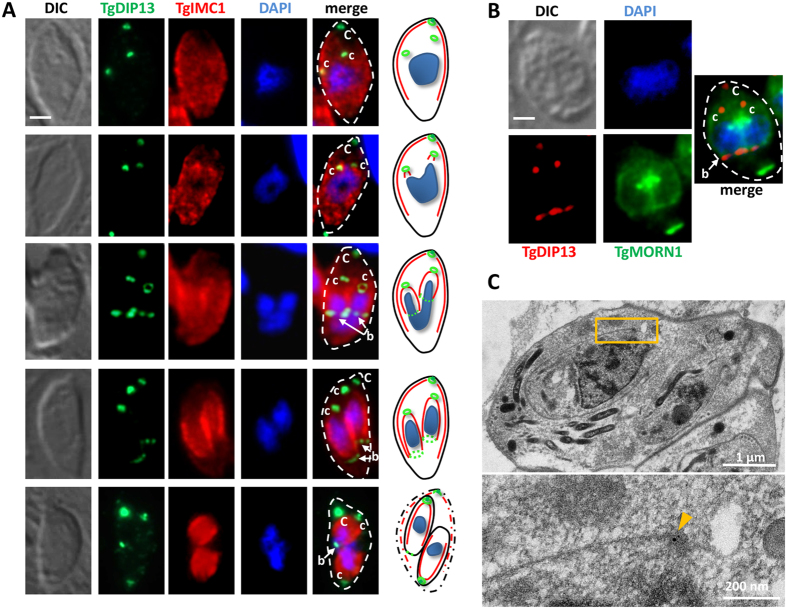
During cell division, TgDIP13 localises to the basal complex of developping daughter cells. (**A**) Co-staining of HA-tagged TgDIP13 and TgIMC1, used as a marker for daughter IMC budding. DNA was labelled with DAPI. C (capital): mother cell conoid; c (lower case): daughter cell conoid; b: basal complex. DIC: differential interference contrast. Parasite shape is outlined on the merged images. Scale bar represents 1 μm. A cartoon schematic describing the progess of division is shown on the right of the panels. (**B**) Co-staining of HA-tagged TgDIP13 and TgMORN1 in dividing parasite shows co-localisation at the basal complex. DNA was labelled with DAPI. C (capital): mother cell conoid; c (lower case): daughter cell conoid; b: basal complex. DIC: differential interference contrast. Parasite shape is outlined on the merged image. Scale bar represents 1 μm. (**C**) Immunoelectron microscopy on developing daughter cells (top) shows HA-tagged TgDIP13 localises at the posterior edge of the IMC, pointed by the arrowhead in magnified image (bottom).

We co-stained parasites for TgDIP13, together with IMC marker TgIMC1 to visualise the formation and elongation of daughter buds (Fig. 4A). We detected the presence of TgDIP13 before IMC budding, confirming the protein is recruited early to the nascent conoid. Later, as daughter cells extended their IMC, the apical TgDIP13 signal was clearly visible as a ring. However, more unexpectedly, TgDIP13 was also found localising to the posterior edge of the extending IMC. This part of nascent daughters contains a ring-like structure named the basal complex, capping the growing ends of the daughters and then constricting to eventually cap the basal pole of mature parasites^8^,^12^,^13^,^14^. We co-stained dividing parasites for TgDIP13 and a protein named ‘membrane occupation and recognition nexus 1’ (TgMORN1) (Fig. 4B), which is a major marker of the basal complex, although it also localises to the spindle poles and the apical complex^8^,^12^,^13^. The TgDIP13 and TgMORN1 signals were found to co-localise to a great extent at the basal complex, confirming TgDIP13 could be part of this structure. However, whereas TgMORN1 remains associated with the basal complex that caps the posterior end of mature parasites, it seems TgDIP13 is only present at this structure during elongation of the daughter cells (Fig. 4A,B). TgDIP13 association with the posterior end of the extending IMC in nascent daughter cells was confirmed by immuno-electron microscopy, using anti-HA antibodies to detect the tagged protein (Fig. 4C). Thus, altogether, our data suggest TgDIP13 has a specific function at the basal complex during daughter cell formation.

### Association of TgDIP13 with the cytoskeleton

**Figure 5:**
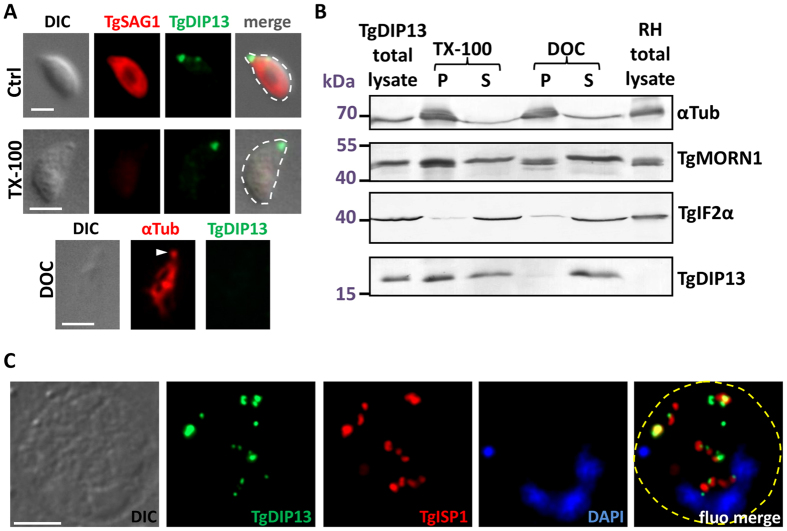
Interaction of TgDIP13 with the cytoskeleton. (**A**) Top: extracellular parasites were extracted or not (Ctrl) with 1% Triton X-100 for 10 minutes and then fixed and co-stained for membrane-associated surface protein TgSAG1 and HA-tagged TgDIP13. Bottom: extracellular parasites were extracted with 10 mM deoxycholate (DOC) for 10 minutes and then fixed and co-stained for α-tubulin (revealing microtubules of the subpellicular cytoskeleton and the conoid (arrowhead)) and HA-tagged TgDIP13. DIC: differential interference contrast. Parasite shape is outlined on the merged images. Scale bar represents 2 μm. (**B**) Parasites were extracted by detergents as described in (**A**) and separated by centrifugation into pellet (P) and soluble (S) fractions. Extracts were subjected to SDS-PAGE, blotted and probed with antibodies. TgIF2α served as a fully soluble protein control. (**C**) HA-tagged TgDIP13-expressing parasites were treated for 24 hours with 2.5 μM oryzalin to disrupt microtubules, and stained For TgDIP13 and TgISP1. Note multiple TgDIP13 and TgIMC1 associated structures. The parasitophorous vacuole membrane is outlined on the merged image. DIC: differential interference contrast. DNA was labelled with DAPI. Scale bar represents 2 μm.

We have seen that TgDIP13 localises to the conoid and, during cell division, to the posterior end of the IMC of developing daughters. Interestingly, shaping of the IMC and its guidance during extension depend on the subpellicular microtubules^27^. Both subpellicular microtubules and the conoid are made of α and β-tubulin dimers^10^. Thus, like its SSNA1/DIP13 eukaryotic counterparts, TgDIP13 localises to microtubule-dependent cytoskeletal structures. We sought to verify the association of TgDIP13 with the parasite’s cytoskeleton by performing detergent extraction and co-fractionation assays. TgDIP13 was found to be largely resistant to extraction with Triton X-100, while it was completely solubilised by a harsher treatment with deoxycholate (Fig. 5A,B), quite similarly to TgMORN1 (Fig. 5B). On the other hand, core constituent of the cytoskeleton such as α-tubulin are resistant to extraction with both detergents (Fig. 5B). Resistance to Triton X-100 extraction is a property shared by parasite cytoskeletal proteins and proteins of the IMC^28^, so our data suggest TgDIP13 could be associated with these structures. However, one should also note that it contrasts with the apparently stronger cytoskeletal association of several of the already characterised resident apical complex proteins, such as TgRNG1^24^, TgRNG2^26^ and TgSAS6L^29^, that have been shown to be at least partly resistant to deoxycholate extraction.

Intracellularly-growing tachyzoites fail to form daughter parasites when they are incubated with oryzalin, a protozoan-specific microtubule-disrupting compound that does not affect host cell cytoskeleton^27^. We thus treated parasites with 2.5 μM oryzalin to block the formation of both spindle and subpellicular microtubules^5^: this prevents daughter budding and results in an undivided, amorphous mother cell with a polyploid DNA content (Fig. 5C). As previously described^23^, in these conditions we failed to observe bud extension with TgIMC1 labelling (not shown), although a ring-like TgISP1 signal likely representing failed attempts to build new daughter buds was observed. Interestingly, a discrete TgDIP13 puncta was found associated to these TgISP1 rings. We conclude that daughter cells microtubules are not required to initiate the formation of the apical TgDIP13 structures in daughter cells, although blocking microtubules elongation prevents further recruitment of the protein to the basal complex.

### TgDIP13 is dispensable for parasite growth *in vitro*

**Figure 6:**
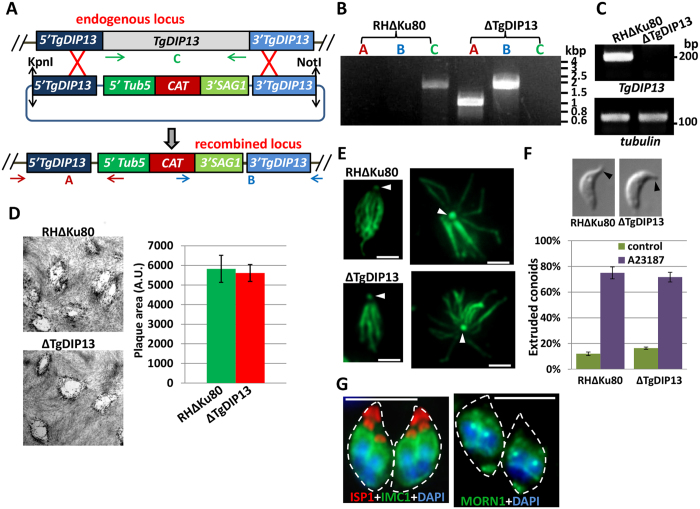
Generation and phenotypic analysis of a *TgDIP13* null mutant. (**A**) A ΔTgDIP13 knock-out cell line was generated in the RHΔKu80 background by double homologous recombination. Clones were obtained after chloramphenicol selection. Arrows represent primers used to verify integration by the PCR shown in (**B**). CAT: chloramphenicol acetyl transferase, 5′Tub5 and 3′SAG1: untranslated regions from the *tubulin* and *SAG1* genes, respectively, for driving *CAT* expression. (**B**) Genomic DNA regions from RHΔKu80 and ΔTgDIP13 cell lines were amplified with primers couples depicted in (**A**) for PCR detections of the endogenous and recombined loci. (**C**) Semi-quantitative RT-PCR analysis of *TgDIP13* expression in the mutant and parental cell lines. Specific *β-tubulin* primers were used as controls. (**D**) Plaque assays were carried out by infecting HFF monolayers with RHΔKu80 or ΔTgDIP13 parasites for 7 days (left). Measurements of lysis plaque areas (right) show no significant defect in ΔTgDIP13 lytic cycle compared to parental cell line. AU: arbitrary units. Values are the mean ± SEM from n = 3 experiments. (**E**) DOC extraction of the subpellicular cytoskeleton, labelled with α-tubulin, shows no visible defect for the subpellicular microtubules or the conoid (arrowhead). Scale bar represents 2 μm. (**F**) Conoid extrusion assays were performed on extracellular parasites using 4 μM of calcium ionophore A23187. No difference was seen in the percentage of extruded conoids in the mutant and control cell lines. Values are the mean ± SEM from n = 3 experiments. DIC images are representative of extruded conoids in parasites of the two cell lines. (**G**) Imaging of dividing ΔTgDIP13 parasites with TgISP1 and TgIMC1 (left) and TgMORN1 (right) shows normal budding of daughter cells. Parasite shape is outlined. DNA was labelled with DAPI. Scale bar represents 5 μm.

The multiple subcellular locations for TgDIP13 at the apical complex of mature parasites and basal complex of developing daughters suggest two separate functions for the protein. To get insights into these potential functions, we generated a TgDIP13 knockout cell line (ΔTgDIP13) by double homologous recombination (Fig. 6A). The replacement of the entire *TgDIP13* coding region by a chloramphenicol acyltransferase (*CAT*) selection cassette was confirmed by PCR (Fig. 6B). Accordingly, semi-quantitative RT-PCR analyses using *TgDIP13-*specific primers showed there was no more *TgDIP13* transcript in the ΔTgDIP13 cell line (Fig. 6C).

The parasite lytic cycle, evaluated *in vitro* by plaque assay on host cells monolayers, was not significantly affected by TgDIP13 depletion (Fig. 6D). As the protein localises to α/β-tubulin-dependent structures, we prepared deoxycholate-extracted parasite microtubule cytoskeleton samples and found that both conoid and the subpellicular microtubules looked normal in the mutant cell line (Fig. 6E). We next checked if depletion of TgDIP13 had affected conoid mobility by performing calcium-dependent conoid extrusion assays, and found no particular phenotype (Fig. 6F). Finally, because the localisation of the protein during cell division, we used IFA with IMC and TgMORN1 labelling to evaluate daughter budding and the structure of the basal complex during the process, respectively, in the mutant cell line. However, nothing odd was detected in the shape of the parasites or the process of daughter formation (Fig. 6G). Overall, this showed TgDIP13 is dispensable for normal *in vitro* growth of tachyzoites.

### TgDIP13 is able to self-associate to form filaments

**Figure 7:**
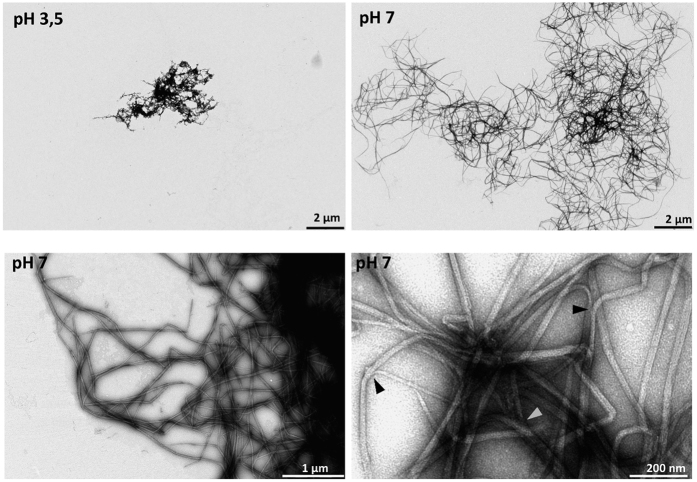
TgDIP13 self-associates to form filaments. Transmission electron microscopic images after uranyl acetate staining of His-tagged recombinant TgDIP13 following incubation of the protein in acidic or neutral pH buffer overnight at 37 °C. Different magnifications are displayed. Examples of branching in the filaments are shown by arrowheads.

A previous investigation on *T. brucei* DIP13 showed this coiled coil protein can polymerise in a head-to-tail arrangement to form long rope-like filaments^17^. Its *T. gondii* homologue, however, possesses a larger disordered C-terminal extension (Fig. 1B). We have nevertheless already shown recombinant TgDIP13 can form dimers (Fig. 1D) and we then sought to investigate if its homo-oligomerisation could lead to higher complexity structures. We incubated purified recombinant TgDIP13 in buffers with acidic or neutral pH, at 37 °C overnight, before analysis by negative stain electron microscopy. TgDIP13 was clearly able to self-assemble into filament-like structures of several dozen μm long and about 20 to 40 nm wide (Fig. 7). The protein formed a complex network, where filaments’ branching was observed, and self-assembly seemed favored at neutral, over acidic, pH (Fig. 7).

### Overexpression of TgDIP13 in the parasites is detrimental and leads to cell cycle defects

**Figure 8:**
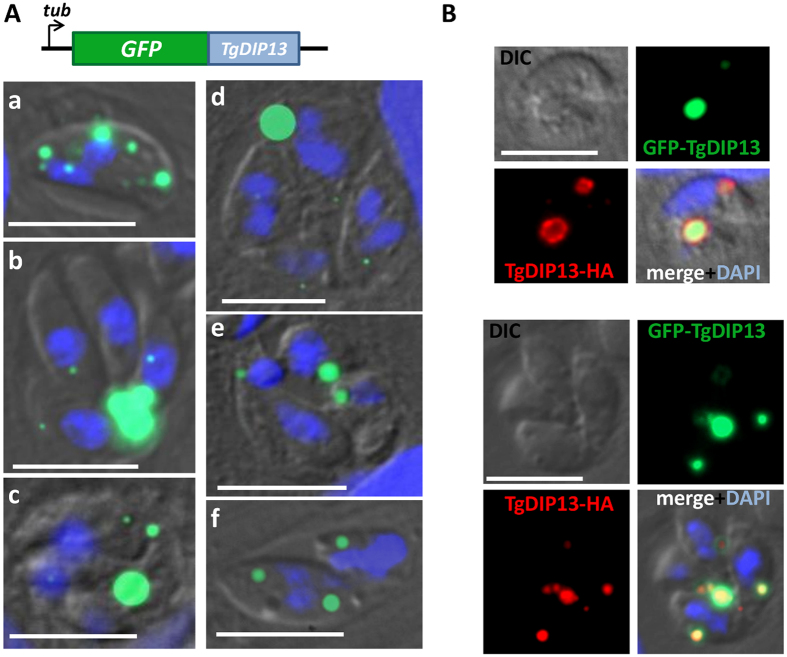
Overexpression of GFP-TgDIP13 perturbs parasite cell division. (**A**) TgDIP13 was fused with GFP at its N-terminal end and overexpressed transiently in parasites under the control of a strong *β-tubulin* promoter. a-f Show representative images of protein aggregates and cell cycle perturbations observed in these parasites. DNA was labelled with DAPI. DIC: differential interference contrast. Scale bar represents 5 μm. (**B**) Parasites stably-expressing a HA-tagged version of endogenous TgDIP13 were transfected to transiently overexpress GFP-TgDIP13. Co-staining revealed endogenous TgDIP13 is recruited to GFP-TgDIP13 structures. DNA was labelled with DAPI. DIC: differential interference contrast. Scale bar represents 5 μm.

As the depletion of TgDIP13 did not provide us with any particular clue on its cellular function, we next decided to try an overexpression strategy. We transfected parasites with a plasmid ectocopy for expressing a N-terminally tagged version of TgDIP13 (GFP-TgDIP13) under the dependence of a strong tubulin promoter (Fig. 8A). We failed to isolate a stable transfected cell line based on the selective marker carried by the plasmid, despite repeated attempts (four independent transfections), suggesting the expression of this construct was lethal for the parasites. In parasites transiently-expressing the fusion protein, observed 24 or 48 hours after transfection, we could see GFP-tagged TgDIP13 was generally mislocalised and formed what looked like large aggregates instead. Aggregates of various sizes were accumulating in some parasites prior to cytokinesis (Fig. 8A,a), while other parasites managed to expel most of the protein in the residual body found in the parasitophorous vacuole after division (Fig. 8A,b). Numerous examples of vacuoles with GFP-TgDIP13-expressing parasites showing problems in the cell division process were observed (Fig. 8A,c–h). Although their nuclei seemed to be able to duplicate normally, these vacuoles contained odd parasite numbers or had smaller or abnormally-shaped parasites. Overall, this suggests mitosis was essentially not affected, but that defects appeared rather at the level of the elongation of newly formed daughters, or at a later stage like cytokinesis. We hypothesize the abnormal localisation and formation of aggregates for GFP-fused TgDIP13 is due to its overexpression and its intrinsic propensity to self-associate, as demonstrated before. For instance, we also tried to overexpress the protein in mammalian cells, this time with a C-terminal myc tag and, quite similarly, it formed very large spherical aggregates which were several μm wide (not shown).

Overexpressed GFP-fused TgDIP13 does not seem to localise to the subcellular compartments where the native protein is usually found, it is thus possible that the detrimental effects of GFP-TgDIP13 overexpression is independent from its cellular functions. To investigate this, we transfected the construct to express GFP-TgDIP13 in the cell line stably-expressing TgDIP13-HA from the endogenous locus. Interestingly, in transfected parasites TgDIP13-HA localised to the GFP-TgDIP13 aggregates, away from its normal subcellular localisation (Fig. 8B). This suggested the overexpressed protein is able to segregate the natively-expressed one, probably because of their ability to associate together.

## Discussion

The cytoskeleton is a key player for cell organisation, motility and division, and has played a major role in the evolution and complexification of eukaryotic cells. The microtubule-dependent cytoskeleton is dynamically and spatially coordinated by MTOCs, which are, in animal cells, centriole-based structures such as the centrosomes or basal bodies at the origin of cilia and flagella. Homologues of SSNA1, a major antigen of a human autoimmune disease whose precise function is currently unknown^15^, have been recently described in several phylogenetically-distant eukaryotic species (green algae and trypanosomes, where it is named DIP13) and found to be associated with centriole/basal body-dependent structures^16^,^17^. Our own homology-based searches detected SSNA1 homologues in a wide array of eukaryotic lineages, which have in common the presence of centriole-based structures sometime during their life cycle.

Interestingly, we found that TgDIP13, the *Toxoplasma* SSNA1/DIP13 homologue, localises primarily to the apical complex of the tachyzoite stage. In many unicellular eukaryotes, polarised cell shape is typically sustained by an orientated submembrane pellicle of microtubules, whose polarity is defined by the basal body or flagellum. *T. gondii* tachyzoites lack a flagellum, but these very polarised cells possess the APR, an original MTOC that surrounds the conoid at the apex of the parasite and plays a similar role. The fact that we localise TgDIP13 on both anterior and posterior sides of the conoid would fit with a phylogenetically-conserved function for SSNA1 homologues as accessory proteins of cellular MTOCs. However it should be noted that, except in the early stages of cell division, we did not find any obvious close association of TgDIP13 with the centrosomes, the other MTOC of *T. gondii* tachyzoites.

Assembly of the new apical complex commences as one of the earliest events of cell replication. Consistent with this, TgDIP13 appeared early in daughter cell formation: after centrosome duplication, but before any extensive elongation of the IMC. We also found TgDIP13 is a dynamic component of the cell division apparatus, as it associates with the basal complex in budding daughter cells. It is similar to TgCEN2, TgMORN1 and dynein light chain (TgDLC), which are basal complex proteins but also components of the apical complex (and the centriole/spindle pole assembly)^8^,^12^,^13^, with the notable difference that these remain associated to the basal complex of mature parasites, whereas TgDIP13 only remains at the apical complex upon completion of daughter cells assembly. During early stages of the division process, both apical and basal complexes originate closely one from another in the vicinity of the centrosomes, and subsequently the basal complex migrates to the basal pole and constricts in the mature parasite^13^,^30^. Although the basal complex contains a number of putative microtubule-binding proteins such as TgDLC and TgCEN2 and the cytoskeleton scaffold presumably grows concurrently from the apical end in the posterior direction, no tubulin-based structures have been observed there. Members of the SSNA1/DIP13 family have been suggested to bind microtubules^16^. Detergent extractions and fractionation experiments showed that TgDIP13 behaves globally as a cytoskeleton-associated protein, although it does not seem to be strongly connected to the microtubule cytoskeleton, as it can be extracted with deoxycholate. Moreover, we have shown that the initiation of TgDIP13-positive structures during daughter bud formation was essentially independent from the integrity of the microtubule cytoskeleton.

In fact, TgDIP13 was found to display remarkable structural properties that allow its self-organisation into higher order structures, in the fashion of intermediate filaments proteins. Like the other members of the SSNA1/DIP13 family^15^, it is predicted to be essentially made of an α-helix potentially able to arrange into a homodimeric coiled coil. Strikingly, the recombinant protein was able to form filament-like structures *in vitro*, as described for its *T. brucei* counterpart^17^. Coiled coil domains are known to be able to form rod-like tertiary structures and assemble into dynamic fibers, meshworks and scaffolds to perform a structural role^31^. A number of centrosomal and other MTOC proteins are predicted to adopt a coiled coil tertiary structure, which may be well-designed for the organisation of multiprotein scaffolds that can increase the local concentration of molecular components, limit nonspecific interactions, and provide spatial control for regulatory pathways in microtubule nucleation^32^. It is interesting to note that a number of conoid-associated proteins, like TgICMAP1^33^ and TgCAP1^34^, are coiled coil proteins.

The TgDIP13-depleted parasites we generated were viable *in vitro* and showed no particular defect in the global architecture of the conoid and the supellicular cytoskeleton, or in daughter cell development. Although there is no TgDIP13 isoform detectable in the *T. gondii* genome, it is possible a yet uncharacterised protein with a similar fold has a redundant function and can complement for TgDIP13 deficiency. In sharp contrast, transient overexpression of GFP-fused TgDIP13 caused the formation of large protein aggregates in tachyzoites, and these parasites showed a significant defect in parasite cell division, leading to the formation of vacuoles with odd parasite numbers or smaller and abnormally shaped parasites. We believe the overexpression of TgDIP13, which is able to self-associate, led to the formation of these aggregates. In a normally-growing population, the punctate or fiber-like TgDIP13 signals we sometimes see in addition to the apical complex localisation, might reflect occasional overexpression of the native protein in the parasites. This suggests TgDIP13 levels have to be tightly regulated for it to reach its normal subcellular localisation and play its normal role. It is also possible that the detrimental effects of GFP-TgDIP13 overexpression are unspecific, and that a large accumulation of the protein would simply physically impair the normal cell division process. However, the parasites seemed to be able to expel large amounts of the protein in the residual body left after cytokinesis, and the parasites displaying the more pronounced phenotype were not necessarily those with the larger aggregates. Instead, we could show that overexpressed GFP-TgDIP13 is able to segregate native TgDIP13, possibly thanks to the self-association properties of the protein. Besides its α-helical domains, TgDIP13 possesses a C-terminal extension compared with other eukaryotic homologues, which might play a role for interactions with parasite-specific proteins. Thus, to explain the dominant-negative phenotype, we postulate segregation of native TgDIP13 by the overexpressed GFP-fusion would also lead to sequestration of one or several binding partners that are important for *Toxoplasma* division. Interestingly, TgMORN1, which also localises to the basal complex, plays a key role in cell shape integrity during daughter cells biogenesis^35^. However, there is so far no evidence of a direct TgDIP13/TgMORN1 interaction, as TgDIP13 was not found in co-immunoprecipitation^36^ or two-hybrid^37^ analyses of TgMORN1 binding partners. A variety of appendages and accessory structures associated with MTOCs are performing roles in the orchestration of cell architecture, and in defining MTOC connections for biogenesis and inheritance. We propose TgDIP13 is a coiled coil protein with scaffolding properties that would have a similar function for parasite-specific cytoskeletal structures.

The conoid is a structure found in *Toxoplasma* and other members of the coccidian subgroup of apicomplexan parasites, as well as in some gregarines, but is thought to be missing from other members of the phylum, such as *Plasmodium*, probably because of a secondary loss from these lineages^38^. The presence of TgDIP13 homologues in members encompassing the whole Apicomplexa phylum confirms the function of this protein is not exclusively linked to this organelle. Its function at the basal complex is more likely to be conserved: *Plasmodium* blood stages, for instance, also display a similar structure during schizogony, which is labelled with PfMORN1^39^,^40^. Interestingly, MORN1 also localises near the flagellar basal bodies in mature microgametes of *T. gondii*^39^. Indeed, in *Toxoplasma* or other Apicomplexa like *Plasmodium*, for example, microgametes stages have a flagellum, although a number of conserved centriolar or flagellar components were apparently lost from the apicomplexan lineage^22^. Human and *Chlamydomonas* homologues of TgDIP13 have been associated with flagellar structures^16^, so it would certainly be interesting to investigate DIP13 function and localisation in flagellated apicomplexan stages. Moreover, although the tachyzoite stage we used in this study lacks a flagellum, it retains several flagellar components that are important for organising cell division such as elements of the flagellar rootlet^41^. It appears that apicomplexan parasites, which had a flagellated algal ancestor and subsequently lost the flagellum for most of their developmental stages, have nevertheless kept the organising principle of the flagellar MTOC, and the conoid has been suggested to derive from a vestigial flagellum^29^. More generally, there have been recent evidences that conserved eukaryotic MTOC-associated proteins can associate with parasite-specific structures. TgCEN2^8^ and TgSAS6L^29^, two proteins typically implicated with centriolar function, are associated with the PCR in *Toxoplasma* tachyzoites for instance. TgDIP13 is a new striking example of a widely conserved eukaryotic protein that has evolved a specialised function dedicated to peculiar cytoskeletal structures in these early-diverging eukaryotes.

## Materials and Methods

### Parasites and cells culture

Tachyzoites of the RHΔKu80^42^ *T. gondii* strain, as well as derived transgenic parasites generated in this study, were maintained by serial passage in human foreskin fibroblast (HFF, purchased from the American Type Culture Collection, CRL 1634) cell monolayer in Dulbecco’s modified Eagle medium (DMEM, Gibco) supplemented with 5% decomplemented fetal bovine serum (Gibco), 2 mM L-glutamine (Gibco) and a cocktail of 100 μg/mL penicillin-streptomycin (Gibco).

### Bioinformatic analyses

Sequence alignments were performed using the MUltiple Sequence Comparison by Log-Expectation (MUSCLE) algorithm of the Geneious software suite (http://www.geneious.com). The phylogenetic tree was built using the Neighbor-Joining method included in the same software suite. Secondary structure predictions were made using the EMBOSS tool garnier (http://www.bioinformatics.nl/cgi-bin/emboss/garnier). Predictions for coiled coils and their oligomerisation state were made using Multicoil (http://groups.csail.mit.edu/cb/multicoil/cgi-bin/multicoil.cgi).

### Generation of an HA-tagged TgDIP13 cell line

To tag endogenous TgDIP13, a triple influenza hemagglutinin (HA) tag was inserted at the C-terminal endogenous locus using a ligation-independent strategy^42^. An 1,1 kpb genomic fragment corresponding to the 3′ end of *TgDIP13* was amplified by PCR with the Phusion polymerase (New England BioLabs) using primers ML2394/ML2395 (see Table S1 for a list of primers used in this study) and inserted in the pLIC-HA_3_-CAT plasmid. The resulting vector pLIC-TgDIP13-HA_3_-CAT was linearised with XcmI and 40 μg were transfected into the TATi1-ΔKu80 cell line to allow single homologous recombination. Transgenic parasites were selected with 20 μM chloramphenicol and clones were isolated by limiting dilution in a 96-well plate.

### Generation of a TgDIP13 knock-out cell line

The TgDIP13 knock-out (ΔTgDIP13) cell line was generated by replacing the endogenous *TgDIP13* locus with a chloramphenicol acetyltransferase (CAT) cassette using double homologous recombination strategy. Briefly, 0.9 kb and 1.1 kb DNA fragments corresponding to regions upstream of the initiation codon and downstream of the stop codon of *TgDIP13*, respectively, were amplified from genomic DNA using primers ML2512/ML2513 and ML2514/ML2515. These fragments were respectively cloned into KpnI/HindIII and BamHI/NotI sites of the pTub5/CAT plasmid^43^. The resulting pTub5/CAT-TgDIP13 plasmid was then digested with KpnI/NotI and transfected into the RHΔKu80 cell line. Transfected parasites were selected with chloramphenicol at concentration of 20 μM and cloned by limit dilutions. Correct integration was verified by PCR in the positive clones (see Fig. 6A,B). Primer couples that were used are, for PCR ‘A’ ML2549/ML2233, for PCR ‘B’ ML388/ML2250, for PCR ‘C’ ML2248/ML2249. RT-PCR detection of mRNA (Fig. 6C) was performed as described before^44^, with primers ML2581/ML2582 for *TgDIP13*, and ML841/ML842 for *β-tubulin*.

### Production of recombinant TgDIP13

*T. gondii* cDNA was used to amplify the *TgDIP13* gene using primers ML2448/ML2449, which was then cloned into NdeI/XhoI-digested pET24a+ (Novagen). The construct was transformed into *E. coli* BL21 cells to produce a recombinant protein with a C-terminal 6 His tag, and further purified on a nickel affinity column in the presence of 6 M urea.

### *In vitro* polymerisation of recombinant TgDIP13

Peak elution fractions of purified recombinant TgDIP13 were pooled and protein concentration was determined using bicinchoninic acid assay (Interchim) before acetone precipitation. The protein pellet was redissolved in a buffer with 50 mM Tris HCl pH 7.5, 50 mM NaCl, 5 mM EDTA and 6 M urea to give a protein concentration of 10 mg/ml. This solution was diluted 1:10 in either 30 mM MOPS pH 7.0, or 0.1 M acetate buffer pH 3.5, and incubated for 24 hours at 37 °C. These solutions were then diluted 1:10 again to a final protein concentration of 0.1 mg/ml before the samples were processed for electron microscopy analysis.

### Electron microscopy

For transmission electron microscopy and negative staining of oligomerised recombinant TgDIP13 protein, samples were processed as described previously^17^. A 8 μl drop of suspension was placed on formvar-carbon-coated grids for 2 min at room temperature (RT), briefly rinsed with water, and stained for 10 min at RT in the dark with 1% uranyl acetate in water.

For immunoelectron microscopy imaging of HA-tagged TgDIP13 at the conoid, infected fibroblast monolayers were grown in cell culture Petri dishes, fixed 1h at room temperature in PBS containing 4% paraformaldehyde and 0,005% glutaraldehyde, and then extracted with 1% Triton X100 in PBS for 10 min. After washing, fresh fixative was applied and samples kept at 4 °C. Samples were incubated 15 min. with PBS 0,05 M glycine, rinsed in PBS and scraped with a piece of Teflon in PBS containing 2% gelatin. Cells were pelleted and embedded in 12% gelatin, cut in small blocks (<1 mm) and infused 24 h in 2.3 M sucrose on a rotating wheel; blocks were mounted on specimen pins and frozen in liquid nitrogen. Cryo-sectioning was performed on a Leica UCT cryo-ultramicrotome, 80 nm cryosections were picked-up in a 1:1 mixture of 2.3 M sucrose and 2% methylcellulose in water and stored at 4 °C. For on-grids immunodetection, grids were floated twice 2 min on water to remove methylcellulose/sucrose mixture, then blocked with 2% skin-fish gelatin (SFG, Sigma) in PBS for 5 min. Successive incubation steps were performed on drops as follows : 1) rat monoclonal anti-HA (clone 3F10, Roche) in 2% SFG, 2) rabbit polyclonal anti-rat IgG antibody (Sigma) in PBS 0.1% bovine serum albumin (BSA), 3) Protein A-gold (UMC) in PBS 0.1% BSA. Four 2 min washes in PBS 0.1% BSA were performed between steps. After Protein A, grids were washed 4 times 2 min. with PBS, fixed 5min in 1% glutaraldehyde in water then washed 6 times 2 min with water. Grids were then incubated with 2% methylcellulose: 4% uranyl acetate 9:1 for 15 min on ice in the dark, picked-up on a wire loop and air-dried.

For immunoelectron microscopy imaging of HA-tagged TgDIP13 at the basal complex, we used pre-embedding. Infected cells were grown on coverslips and fixed as above. Immunodetection was performed as described below for immunofluorescence, except that secondary detection was done using a rabbit polyclonal anti-rat IgG antibody (Sigma), followed by anti-rabbit Alexa 488 fluoro-nanogold (Nanoprobes) in PBS with 1% (w/v) nonfat dry milk. Samples were then post-fixed with 1.25% glutaraldehyde in cacodylate buffer with 5% sucrose for 2 hours at RT. Gold particles enhancement was carried out using the GoldEnhance EM Plus kit (Nanoprobes). Samples were then fixed with 2% osmium tetroxide and 1.5% Potassium ferricyanide, dehydrated in acetonitrile series and embedded in Epon 812. Ultrathin sections were contrasted with uranyl actetate and lead citrate.

Samples were observed on a JEOL 1200 EXII Transmission electron microscope on the EM platform of the University of Montpellier. All chemicals were from Electron Microscopy Sciences unless otherwise mentioned.

### Protein extraction assay and immunoblot analyses

Freshly lysed parasites were collected by centrifugation at 1,000 g for 10 minutes. These samples were suspended in PBS containing 1% Triton X-100, or PBS with 10 mM deoxycholate for 15 minutes at room temperature. For immunoblot analysis, samples were centrifuged at 12,000 g for 30 minutes to separate the pellet from the soluble fraction. The latter was acetone-precipitated and both fractions were resuspended in the same volume of loading buffer before being separated by SDS-PAGE and analysed by immunoblot. The primary antibodies used for detection and their respective dilutions were: mouse monoclonal anti-α-tubulin (clone B-5-1-2, Sigma-Aldrich) at 1/4000, rabbit polyclonal anti-TgMORN1 at 1/1000^12^, rabbit polyclonal anti-TgIF2α at 1/500^45^ and rat monoclonal anti-HA at 1/1000 (clone 3F10, Roche).

Detergent-extracted parasite samples for immunofluorescence were settled onto poly-L-lysine-coated glass slides (Thermo Scientific) for 15 minutes at room temperature. These samples were fixed with 4% (w/v) paraformaldehyde in PBS, prior to processing for immunofluorescence (see below).

### Immunofluorescence microscopy

For IFA, intracellular tachyzoites grown on a monolayer of HFF cells were fixed for 20 min with 4% (w/v) paraformaldehyde in PBS (except for immunostainings involving the anti-TgMORN1 antibody, for which fixation was performed with methanol at −20 °C for 5 minutes), permeabilised for 10 min with 0.3% Triton X-100 in PBS and blocked with 0.1% (w/v) BSA in PBS. Primary antibodies used (at 1/1000, unless specified) for detection of the organelles were: mouse monoclonal anti-TgISP1^23^, rabbit polyclonal anti-TgMORN1^12^, mouse monoclonal anti-TgSAG1^46^, mouse monoclonal anti-α-tubulin (clone B-5-1-2, Sigma-Aldrich) at 1/2000, rabbit polyclonal anti-TgIMC1^47^. Transient transfections of GFP-tagged apical complex markers were used for co-staining: GFP-TgCEN2 and GFP-TgCAM1^8^, and TgRNG1-GFP^26^. Staining of DNAs was performed on fixed cells incubated for 5 min in a 1 μg/ml DAPI solution. All images were acquired at the Montpellier RIO imaging facility from a Zeiss AXIO Imager Z2 epifluorescence microscope equipped with a Camera ORCA-flash 4.0 camera (Hammamatsu) and driven by the ZEN software. Adjustments for brightness and contrast were applied uniformly on the entire image.

### Plaque assays

A confluent monolayer of HFFs grown in 24-well plates was infected with 2 × 10^5^ freshly egressed tachyzoites and incubated for 6 days. The infected cell layer was then rapidly fixed in cold methanol (1 min) and stained with Giemsa (1 min). Images were acquired with an Olympus MVX10 macro zoom microscope equipped with an Olympus XC50 camera. Plaque area measurements were done using Zen software (Zeiss).

### Conoid extrusion assay

Freshly egressed parasites were washed in HEPES buffered saline and incubated for 5 minutes with 4 μM of calcium ionophore A23187 in the presence of 5 mM CaCl_2_. Samples were then fixed immediately with 4% (w/v) paraformaldehyde, settled onto poly-L-lysine-coated glass slides (Thermo Scientific) for 15 minutes at room temperature, and processed for microscopic imaging as described above.

## Additional Information

**How to cite this article**: Lévêque, M. F. *et al*. An evolutionarily conserved SSNA1/DIP13 homologue is a component of both basal and apical complexes of *Toxoplasma gondii*. *Sci. Rep.* **6**, 27809; doi: 10.1038/srep27809 (2016).

## Supplementary Material

Supplementary Information
