# Supplementary material for: An evolutionarily conserved SSNA1/DIP13 homologue is a component of both basal and apical complexes of Toxoplasma gondii
Source: Sci Rep. 2016 Jun 21;6:27809. doi: 10.1038/srep27809 (PMC4914967; doi:10.1038/srep27809)
Supplement: Supplementary Information [file srep27809-s1.pdf]

An evolutionarily conserved SSNA1/DIP13 homologue is a component of both basal and apical complexes of *Toxoplasma gondii*

Maude F. Lévêque, Laurence Berry and Sébastien Besteiro*

DIMNP, UMR5235, CNRS, University of Montpellier, Montpellier, France

*Author for correspondence: sebastien.besteiro@inserm.fr

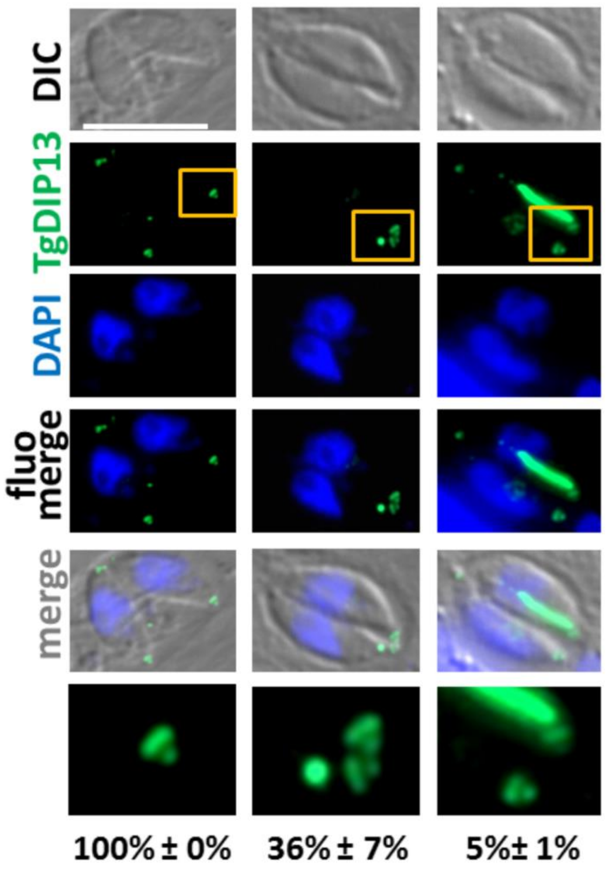

Figure S1. TgDIP13 localises to the apical complex and occasionally displays additional signals. Immunofluorescence detection of HA-tagged TgDIP13 in non-dividing parasites and quantification of the number of parasites displaying an apical signal and additional punctate or fiber-like signals. Magnifications of squared regions are shown at the bottom. DNA was labelled with DAPI. DIC: differential interference contrast. Scale bar represents 5 µm.

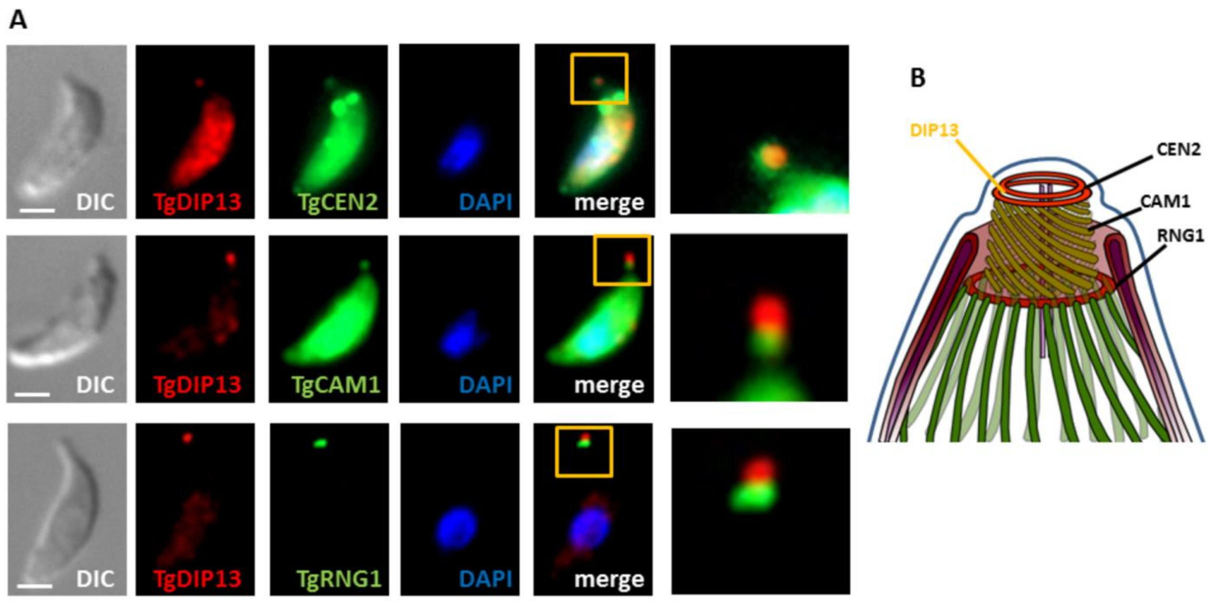

Figure S2. TgDIP13 is associated with the anterior part of the conoid following organelle extrusion. A) Co-staining between HA-tagged TgDIP13 and apical complex markers TgCEN2, TgCAM1 and TgRNG1 on extracellular parasites following conoid extrusion. DNA was labelled with DAPI. DIC: differential interference contrast. Scale bars represent 1 µm. Magnifications of squared areas are shown on the right. **B**) Schematic representation of the localisation of TgDIP13 and other apical complex markers in an extruded conoid conformation.

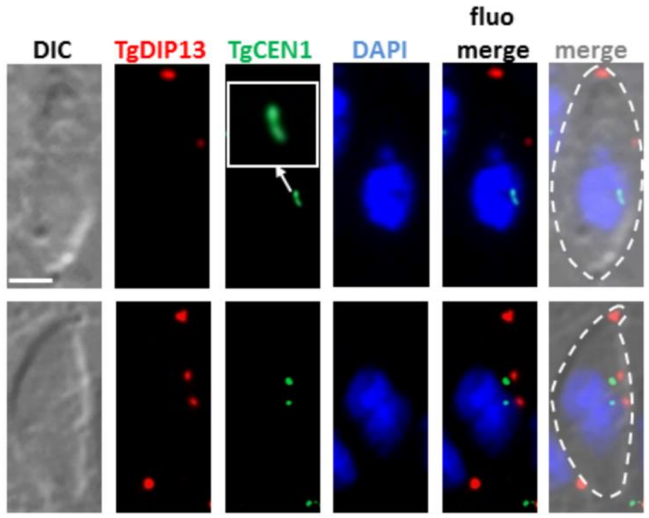

Figure S3. TgDIP13 signal appears in daughter cells after centrosome duplication.

Parasites were co-stained for HA-tagged TgDIP13 and centrin 1. Inset shows magnification of a duplicating centrosome, in the absence of a detectable TgDIP13 signal. Parasite shape is outlined on the merged images. DNA was labelled with DAPI. DIC: differential interference contrast. Scale bar represents 1 μm.

**ML388**	ACAGTACTGCGATGAGTGGTGGC
**ML841**	ATGTTCCGTGGTCGCATGT
**ML842**	TTCATGTTGTTGGGAATCCAC
**ML2233**	TTTCACGAACGCGGACATGC
**ML2248**	CATATGACGAGCACAACGAACTCC
**ML2249**	CTCGAGTCCTGCGCCAGCTGTGGG
**ML2250**	CAGGATGATTCGTCAGTAGG
**ML2394**	TACTTCCAATCCAATTTAATGCTGCAAAAGGAGATCGCG
**ML2395**	TCCTCCACTTCCAATTTTAGCTCCTGCGCCAGCTGTG
**ML2512**	GGAGGTACCGACAAGTCGTCTTG
**ML2513**	GGGAAGCTTCTTGAAAATCGTTCTGG
**ML2514**	GGATCCACGTATCTGTCGGATTTCC
**ML2515**	CCTGCGGCCGCTTTCTAGAATTTTGTTTGG
**ML2549**	TAACTGGTTCAGTCCTGTAG
**ML2581**	CTTACACAACTACAACCACG
**ML2582**	TGAAGCCGTTCCTGCAGAAG

Table S1. Primers used in this study.
